# Alcohol decreases intestinal ratio of *Lactobacillus* to *Enterobacteriaceae* and induces hepatic immune tolerance in a murine model of DSS-colitis

**DOI:** 10.1080/19490976.2020.1838236

**Published:** 2020-11-12

**Authors:** Paulius V. Kuprys, Abigail R. Cannon, Jennifer Shieh, Noama Iftekhar, Sun K. Park, Joshua M. Eberhardt, Xianzhong Ding, Mashkoor A. Choudhry

**Affiliations:** a Alcohol Research Program, Loyola University Chicago Health Sciences Campus, Maywood, IL, USA; b Burn and Shock Trauma Research Institute, Loyola University Chicago Health Sciences Campus, Maywood, IL, USA; c Department of Surgery, Loyola University Chicago Health Sciences Campus, Maywood, IL, USA; d Integrative Cell Biology Program, Loyola University Chicago Health Sciences Campus, Maywood, IL, USA; e Department of Pathology, Loyola University Chicago Health Sciences Campus, Maywood, IL, USA

**Keywords:** *Enterobacteriaceae*, *Lactobacillus*, ethanol, colitis, liver, inflammation, immune tolerance

## Abstract

Alcohol can potentiate disease in a mouse model of dextran sodium sulfate (DSS) colitis; however, the underlying mechanism remains to be established. In this study, we assessed whether the potentiated disease could be related to *Enterobacteriaceae* and *Lactobacillus*, as changes in their relative abundance can impact intestinal health. We also assessed whether the intestinal barrier is compromised after alcohol and DSS as it may increase bacterial translocation and liver inflammation. Mice were administered DSS followed by binge ethanol or water vehicle, generating four experimental groups: (Control+Vehicle, Control+Ethanol, DSS+Vehicle, DSS+Ethanol). DNA was isolated from colon and cecal contents followed by qPCR for levels of *Enterobacteriaceae* and *Lactobacillus*. Colon and liver sections were taken for histology. Intestinal epithelial cells were isolated from the colon for RNA expression. DSS+Ethanol cecal contents exhibited a 1 log increase in *Enterobacteriaceae* (*p* < .05), a 0.5 log decrease in *Lactobacillus*, and a 1.5 log decrease (*p* < .05) in the *Lactobacillus:Enterobacteriaceae* ratio compared to DSS+Vehicle, with similar trends in colon contents. These changes correlated with shorter colons and more weight loss. Irrespective of ethanol administration, DSS compromised the mucosal barrier integrity, however only DSS+Ethanol exhibited significant increases in circulating endotoxin. Furthermore, the livers of DSS+Ethanol mice had significantly increased levels of triglycerides, mononuclear cells, yet exhibited significantly depressed expression of liver inflammatory pathways, suggestive of tolerance induction, compared to mice receiving DSS+Vehicle. Our results suggest that ethanol after DSS colitis increases the intestinal burden of *Enterobacteriaceae* which may contribute to intestinal and liver damage, and the induction of immune tolerance.

## Introduction

Inflammatory bowel diseases (IBD) are highly prevalent within the United States, affecting 1.5 million individuals.^1^ The incidence of IBD in other countries is also increasing rapidly.^1^ There are two main forms of IBD: Crohn’s disease (CD), which produces discontinuous lesions throughout the gastrointestinal tract, and ulcerative colitis (UC), which produces a continuous mucosal lesion that is localized to the colon.^2^ The onset of disease in both CD and UC follows a similar course, whereby patients experience intense abdominal pain, diarrhea, and bloody stools. While the etiology of the disease is not fully understood, studies suggest that genetic, environmental, and microbial factors can contribute to the disease onset.^3^ After the initial onset of disease, individuals with IBD experience cycles of active disease followed by quiescent periods. With regard to UC, the flares in disease are frequent, with 80% of UC patients experiencing a relapse within 2 years of entering remission.^4^ No cure is available, thus maintenance of remission and preventing flares is the current mainstay of treatment. What leads to a flare in the disease is not entirely defined but lifestyle and dietary factors,^3^ such as alcohol,^5^ have been implicated. Studies examining IBD patients have found that alcohol consumption is associated with worsening of gastrointestinal symptoms,^6,7^ induction of flare,^8^ and increased intestinal infections.^9^ These effects may arise from alcohol’s ability to alter the intestinal microbiota,^10–14^ which would negatively impact the already altered intestinal microbiome of IBD patients.^15–17^ In particular, alcohol consumption and IBD are independently characterized by intestinal decreases of the *Lactobacillus* genus,^18,19^ and increases of the *Enterobacteriaceae* family.^14–18,20^ The observed increase in *Enterobacteriaceae* is relevant because they can penetrate the mucus layer of intestines in UC patients,^15,16,21^ and express pro-inflammatory endotoxin.

Murine colitis models show that *Enterobacteriaceae* penetration occurs before the onset of intestinal tissue damage.^22,23^ A decrease in lactobacilli may further compound this effect since they promote intestinal health and also check the growth of *Enterobacteriaceae*.^24–27^ Studies have determined that the intestinal ratio of these two bacteria can be an indicator of gut health,^28,29^ and thus, changes in their relative abundance may disrupt the normal gut homeostasis. This may allow for leakage of gut bacteria and/or their products into systemic circulation, which have been implicated as causal agents of liver inflammation and damage.^30–32^ IBD patients experience higher levels of liver steatosis and altered liver enzymes,^33,34^ which may in part be explained by a combination of increased bacterial translocation and increased *Enterobacteriaceae* load. Using a mouse model of DSS-induced colitis, we explored the effect of ethanol on intestinal levels of *Enterobacteriaceae* and lactobacilli within the context of colitis severity and liver changes.

## Results

### Alcohol after DSS colitis increases Enterobacteriaceae and decreases Lactobacillus in colon and cecal contents

**Figure 1.**
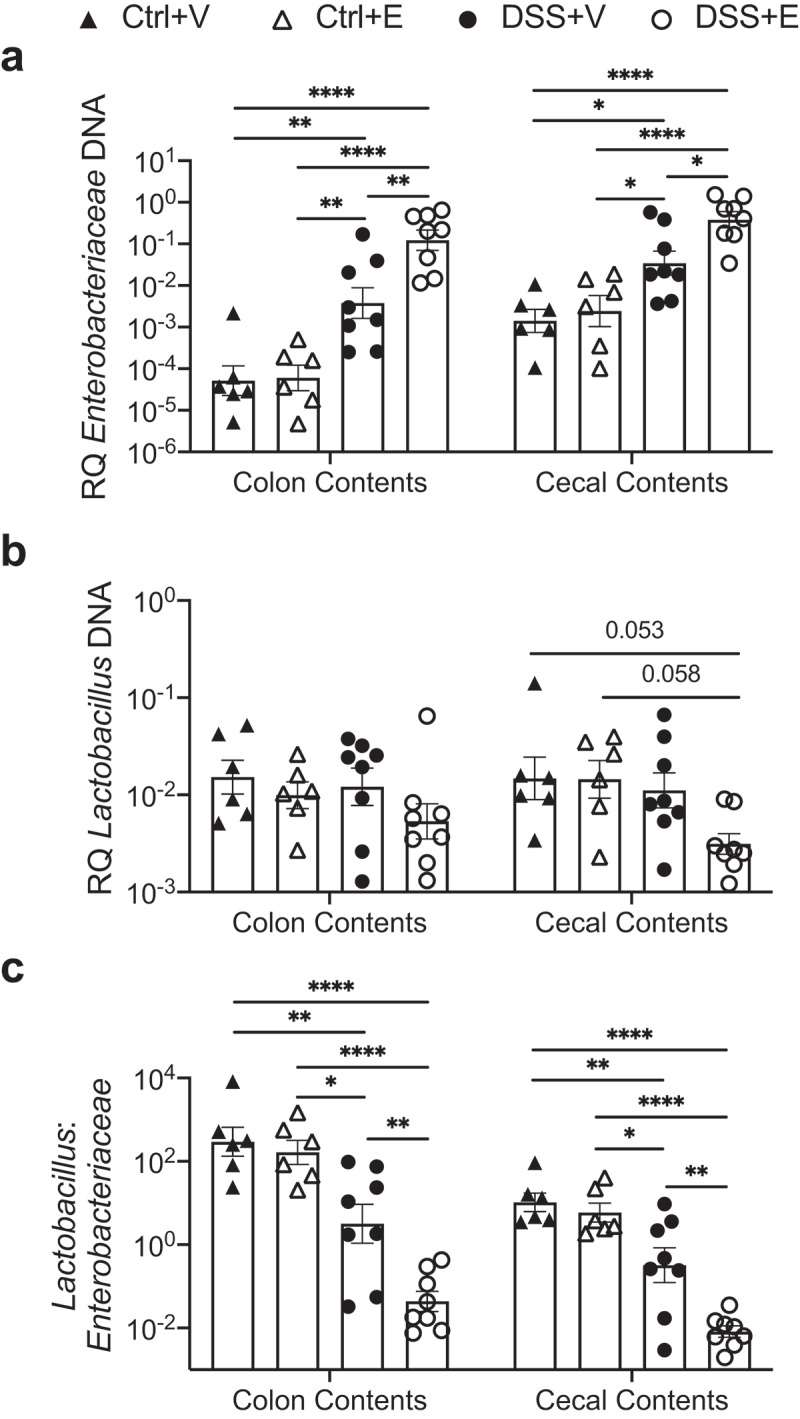
Increased *Enterobacteriaceae* and decreased Lactobacillus in large intestine and cecal contents of DSS+Ethanol mice. Cecal and colon contents were harvested from mice after euthanasia on day 7, 3 hours after gavage, followed by DNA isolation, qPCR, and determination of bacterial RQ for (a) *Enterobacteriaceae*, (b) *Lactobacillus*, (c) *Lactoabacillus:Enterobacteriaceae*. Bars display mean ± SEM, with each symbol representing data from one mouse. RQ: Relative Quantity. Statistics by One-Way ANOVA with Tukey post-hoc test. n = 6–8 per group. * *p* < .05, ** *p* < .01, **** *p* < .0001

Consistent with our previous observations,^9^ mice gavaged with alcohol after DSS treatment shows more pronounced weight loss and shorter colon lengths (Figure S1a–c). To assess intestinal bacterial changes, cecal contents, and colon contents were harvested from mice euthanized on experimental day seven, 3 h after final gavage. DNA was isolated from the colon and cecal contents followed by quantitative PCR (qPCR) to detect relative copy numbers of *Enterobacteriaceae* and *Lactobacillus* 16S rRNA. Both the colon and cecal contents of the DSS administered mice had significantly increased *Enterobacteriaceae* compared to the control mice (*p* < .05; Figure 1a). Furthermore, between the DSS mice, ethanol administration produced a 1 log increase (*p* < .05) in the *Enterobacteriaceae* compared to vehicle administration. *Lactobacillus* levels were decreased 0.5 log in DSS+Ethanol mice compared to all other experimental groups, which was not significant, but a trend for significance was observed in the cecal contents (Figure 1b). Ratios of *Lactobacillus* to *Enterobacteriaceae* have been used as determinants of intestinal health.^28,29^ This ratio is significantly depressed (>1.5 log, *p* < .05) in DSS administered mice compared to control mice (Figure 1c). Meanwhile between the DSS treated groups, ethanol gavage significantly decreases the *Lactobacillus:Enterobacteriaceae* ratio in colon (~1 log, *p* < .05) and cecal (~1.5 log, *p* < .05) contents compared to vehicle (Figure 1c).

### A single ethanol gavage alters Enterobacteriaceae and Lactobacillus levels in fecal pellets

**Figure 2.**
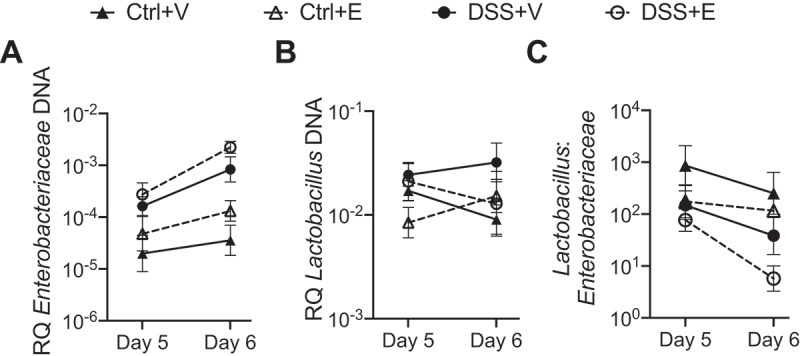
*Lactobacillus:Enterobacteriaceae* decreases in DSS+Ethanol fecal pellets from Day 5 to Day 6. Fecal pellets were collected from mice after weighing and prior to gavage followed by DNA isolation, qPCR, and determination of bacterial RQ for (a) *Enterobacteriaceae*, (b) *Lactobacillus*, (c) *Lactobacillus: Enterobacteriaceae*. Some mice did not produce a fecal pellet, limiting continuous sampling from the same mouse, and therefore data are presented as a summary of each experimental group. Symbols represent the mean ± SEM for the experimental group. RQ: Relative Quantity. n = 4–6 per group

To define the longitudinal changes of the *Enterobacteriaceae* and *Lactobacillus*, we collected fecal pellets from the mice after weighing the mice and before gavaging with vehicle or ethanol on days 5 and 6. The effect of ethanol on bacterial populations in mice receiving combined DSS+Ethanol can be appreciated on day 6, one day after the first administration of alcohol (Figures 2 and S2). From day 5 to day 6, there is a 0.9 log increase in the *Enterobacteriaceae* from DSS+Ethanol mice compared to a 0.7 log increase from the DSS+Vehicle mice (Figure 2a). During this same time period, the *Lactobacillus* decreases by 0.2 log in the DSS+Ethanol mice, while there is a slight increase (0.1 log) in the DSS+Vehicle mice (Figure 2b). This culminates in a~1 log decrease in the *Lactobacillus:Enterobacteriaceae* for the DSS+Ethanol mice, whereas this ratio decreases by ~0.5 log in DSS+Vehicle mice (Figure 2c).

These results suggest that the alcohol administration elicits an effect on the intestinal microbiota after colitis leading to increases in *Enterobacteriaceae* and decreases in *Lactobacillus*, producing a decreased *Lactobacillus:Enterobacteriaceae* ratio. The higher level of *Enterobacteriaceae* and lower level of *Lactobacillus* may contribute to disease flares and increased intestinal inflammation.

### Increased Enterobacteriaceae and decreased Lactobacillus are associated with aspects of disease severity

**Figure 3.**
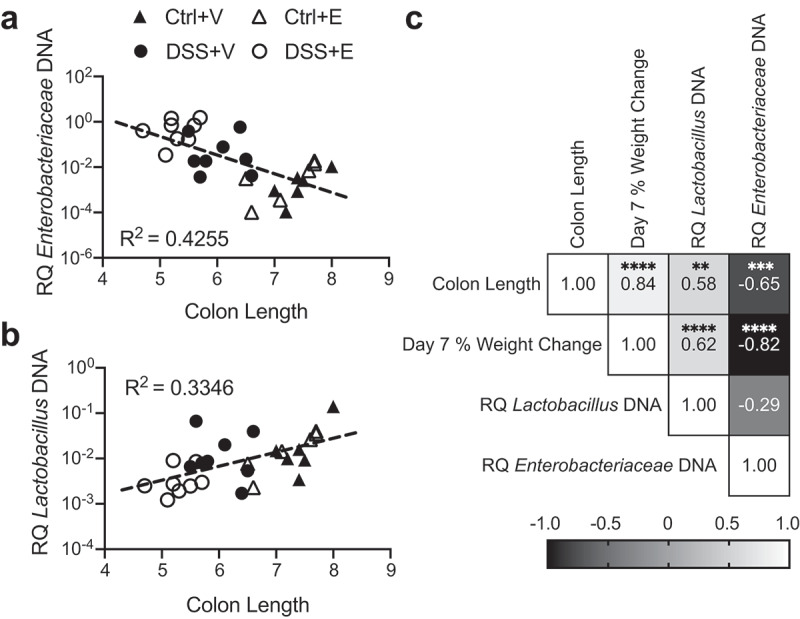
*Enterobacteriaceae* inversely correlates with colon length and *Lactobacillus* positively correlates with colon length. (a) Cecal *Enterobacteriaceae* vs colon length linear regression with corresponding R^2^ value. (b) Cecal *Lactobacillus* vs colon length linear regression with corresponding R^2^ value. (c) Pearson correlation matrix examining correlation between colon length, day 7 percent weight change, cecal *Enterobacteriaceae*, and cecal *Lactobacillus*. Each symbol represents data from one mouse. RQ: Relative Quantity. Statistics by Pearson correlation. n = 6–8 per group. ** *p* < .01, *** *p* < .001, **** *p* < .0001

To further assess how the disease state of these mice relates to the intestinal bacteria, we performed a linear regression of the cecal contents bacteria in relation to the mouse colon length. The colon length is a macroscopic indicator of the inflammatory status of the colon, where longer colons are healthier and shorter colons are more inflamed. In our experiment, the colon length is inversely correlated to cecal content of *Enterobacteriaceae* (Figure 3a) and directly correlated to cecal content of *Lactobacillus* (Figure 3b). On the scatter plot of the linear regression, samples from the DSS+Ethanol mice have shorter colons in conjunction with increased *Enterobacteriaceae* (Figure 3a) and decreased *Lactobacillus* (Figure 3b). In addition, combining data from all four experimental groups, we examined the correlation of colon length, day 7 percent weight change, and cecal contents bacteria using a Pearson correlation (Figure 3c). This revealed that the colon length, and day 7 percent weight change are positively correlated with *Lactobacillus* while being inversely correlated with *Enterobacteriaceae*. Meanwhile, the relationship of *Enterobacteriaceae* and *Lactobacillus* are inversely correlated, although this result was not significant (*p* = .139).

### Intestinal mucin expression is altered post DSS-colitis with no additional effect by ethanol

**Figure 4.**
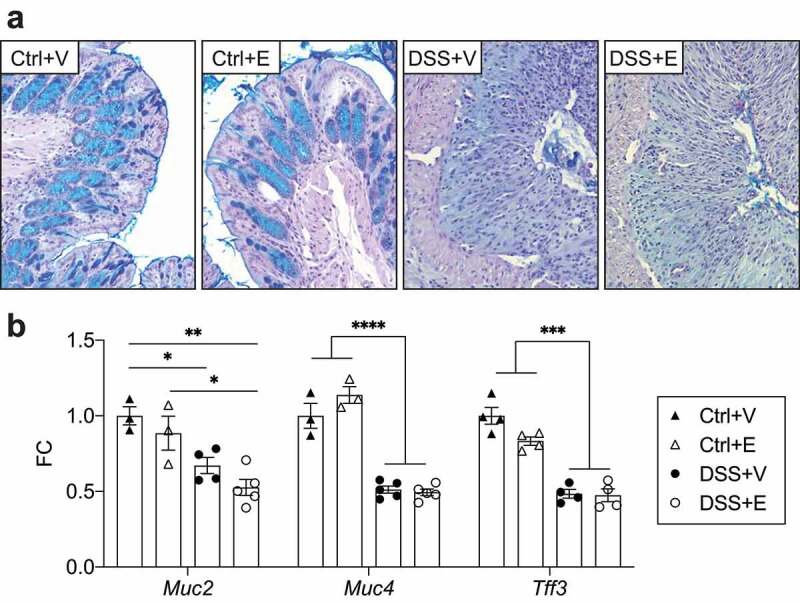
DSS decreases intestinal mucin staining and IEC mucin RNA expression with no additional effect after ethanol. (a) PAS-Alcian blue stained slides of distal colon from mice. Representative of 6–8 mice pre-group. Images taken at 400x total magnification. (b) IEC RNA expression of mucins and intestinal trefoil factor. n = 3–5 per group. Bars display mean ± SEM, with each symbol representing data from one mouse. FC: Fold change. Statistics by One-Way ANOVA with Tukey post-hoc test. * *p* < .05, ** *p* < .01, *** *p* < .001, **** *p* < .0001

The healthy large intestine is covered by mucins, which act as a barrier to limit bacterial interaction with the intestinal epithelium.^35^ However, in IBD there are marked disruptions of the intestinal mucin, which allow for increased interaction of the bacteria with the intestinal epithelium, which may induce a flare.^36–39^ In our model, we first assessed mucin changes using a PAS-Alcian blue stain of intestinal sections. We observed that mucin staining was unaffected in the control mice but severely decreased in the DSS mice regardless of ethanol exposure (Figure 4a). The decreased intestinal mucin levels in the DSS mice suggests that bacterial interaction with the epithelium is uninhibited. We isolated large intestine epithelial cells (IECs) and assessed their expression of mucins and trefoil factor, a marker of intestinal health. Mucin 2, mucin 4, intestinal trefoil factor 3 were all significantly decreased ~twofold in the DSS+Vehicle and DSS+Ethanol mice when compared to the Control+Vehicle mice (*p* < .05) (Figure 4b). This mirrors the histologic changes observed in Figure 4a. These data suggest that DSS induces a primary effect on intestinal mucin depletion, while alcohol has no additive effect.

### Bacterial infiltration of the mucosa, tight junction protein expression, and plasma endotoxin levels

**Figure 5.**
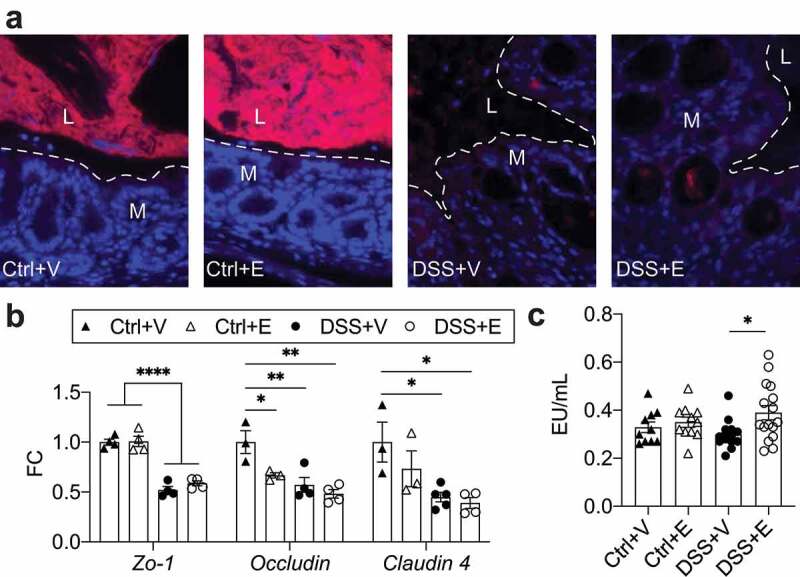
DSS increases bacterial infiltration of intestinal mucosa and decreases expression of tight junction proteins with increases of circulating endotoxin in DSS+Ethanol. (a) Distal large intestine sections displaying localization of bacteria in red. Dashed line represents interface between intestinal epithelium and the intestinal lumen. Representative of 4–6 mice pre-group. Images taken at 400x total magnification. M: Mucosa; L: Lumen. (b) IEC RNA expression tight junction proteins. n = 3–5 per group. (c) Plasma levels of endotoxin. n = 10–16 per group. Bars display mean ± SEM, with each symbol representing data from one mouse. Zo-1: Zonula occludens-1, FC: Fold change. Statistics by One-Way ANOVA with Tukey post-hoc test. * *p* < .05, ** *p* < .01, **** *p* < .0001

To determine bacterial penetration of the epithelial lining, we used fluorescent *in situ* hybridization with a probe targeting all bacteria. We detected an increased presence of bacteria in the mucosa of DSS mice; meanwhile bacteria were largely relegated to the intestinal lumen in control mice (Figure 5a). The presence of bacteria in the mucosa prompted examination of intestinal tight junction proteins. These proteins limit bacterial translocation, and have been found to be decreased in patients with colitis.^40^ IEC RNA expression of tight junction proteins revealed that DSS administration with or without ethanol led to ~twofold decrease in ZO-1, Occludin, and Claudin-4 compared to the Control+Vehicle (*p* < .05) (Figure 5b). Some direct effects of ethanol appear to also occur as there was a significant decrease in Occludin expression in the Control+Ethanol mice compared to the Control+Vehicle mice, which is not significant between the DSS+Vehicle or DSS+Ethanol mice. The combined observations of decreased tight junction expression and increased mucosal infiltration suggest that there is increased opportunity for translocation of bacteria and their by-products, such as endotoxin. We, therefore, assessed plasma endotoxin levels as a marker of translocation. DSS+Ethanol mice exhibited significantly increased plasma endotoxin levels compared to DSS+Vehicle, but were not significantly different when compared to the control groups (Figure 5c). This effect may be secondary to the increased burden of *Enterobacteriaceae* in the DSS+Ethanol mice. As the liver is the primary site of interaction for any translocated bacteria and endotoxin, we next assessed for liver changes in our model.

### DSS+Ethanol mice livers exhibit increased lipid deposition

**Figure 6.**
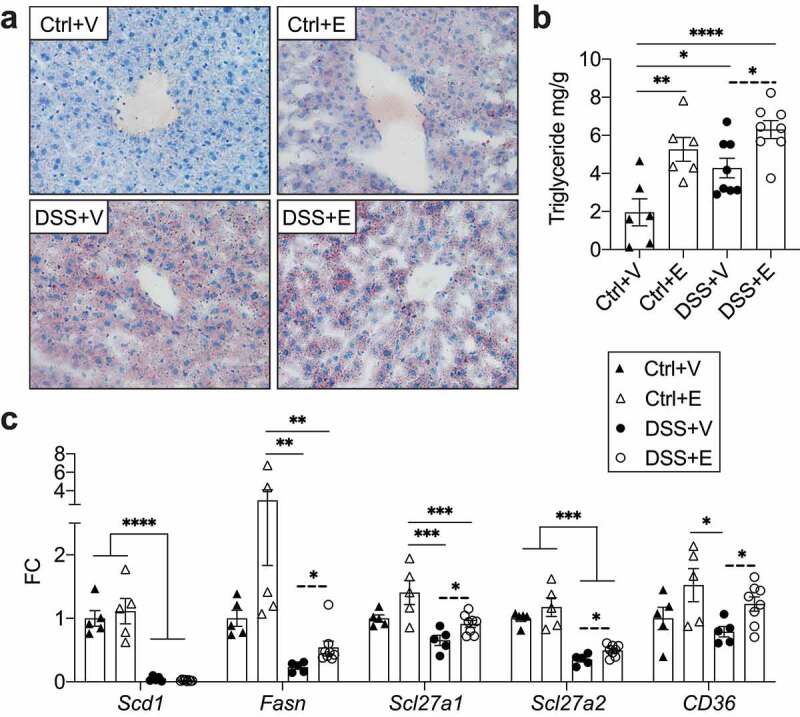
Increased lipid deposition and gene expression associated with lipid synthesis and uptake in DSS+Ethanol livers. (a) Oil Red O stain of frozen liver sections harvested from mice after euthanasia on day 7. Images taken at 400x total magnification. Representative images of n = 6–8 per group. (b) Liver triglyceride mg/g of liver tissue. n = 6–8 per group. (c) Expression of hepatic lipid synthesis and uptake genes. n = 5–8 per group. Bars display mean ± SEM, with each symbol representing data from one mouse. FC: Fold change. Solid lines indicate statistics by One-Way ANOVA with Tukey post-hoc test. Dashed lines indicate significance by student’s t-test. * *p* < .05, ** *p* < .01, *** *p* < .001, **** *p* < .0001

Accumulation of lipids within the liver is a marker of metabolic dysfunction and can be a consequence of alcohol consumption.^41,42^ Qualitative assessment of liver fat deposition using Oil Red O showed increased lipid staining in all experimental groups relative to Control+Vehicle (Figure 6a). In addition, the liver triglyceride concentrations were found to be highest in the DSS+Ethanol mice and this increase was significant when compared to the DSS+Vehicle mice (*p* = .0117, t-test) (Figure 6b). To identify the pathways promoting liver lipid accumulation in our model, we examined the expression of hepatic components involved in lipid synthesis (*Scd1* and *Fasn*) and uptake (*Scl27a1, Scl27a2, CD36*) (Figure 6c). Overall DSS appeared to reduce expression of these components with respect to Control. Meanwhile between DSS+Vehicle and DSS+Ethanol, ethanol significantly promoted the expression of all these components except for *Scd1*. These results indicate that while DSS induces liver lipid deposition, the combination of DSS and ethanol further increases that effect likely through the upregulation of lipid uptake and synthesis pathways.

### DSS+Ethanol mice livers exhibit features of immune tolerance

**Figure 7.**
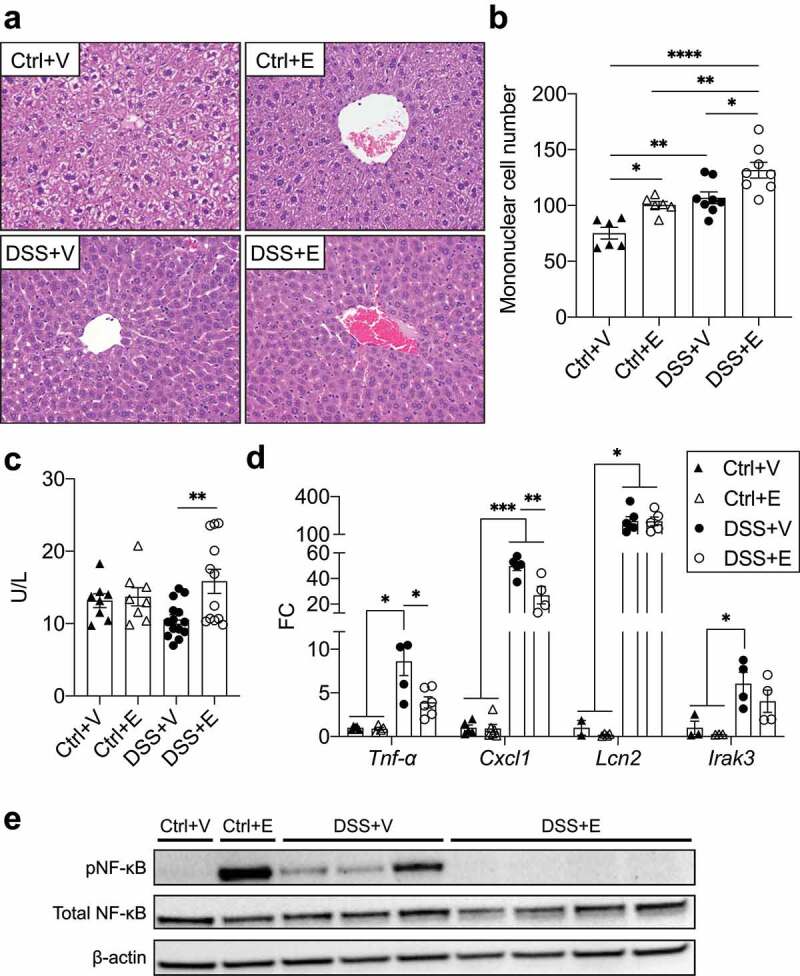
DSS+Ethanol mice exhibit hepatic immune tolerance in the setting of increased plasma ALT. (a) H&E stained liver sections. Taken at 400x total magnification. Images representative of n = 6–8 per group (b) Liver mononuclear cell number counted per liver slide. (c) Plasma levels of ALT. n = 8–14 per group. (d) Liver inflammatory gene expression. n = 3–6 per group. (E) Western blot of liver homogenates for pNF-κB, total NF-κB, and β-actin. Bars display mean ± SEM, with each symbol representing data from one mouse. FC: Fold change. Statistics by One-Way ANOVA with Tukey post-hoc test. * *p* < .05, ** *p* < .01, *** *p* < .001, **** *p* < .0001

Bacteria and bacterial products that translocate from the colon, enter into the mesenteric circulation and can eventually arrive at the liver.^43^ Within the liver, these translocated elements can induce inflammation and accumulation of inflammatory cells.^30^ To assess liver inflammation in our model, we counted the number of mononuclear cells located around the central vein of H&E stained liver sections (Figure 7a). The mean number of mononuclear cells in Control+Ethanol, DSS+Vehicle, and DSS+Ethanol were significantly increased compared to Control+Vehicle (Figure 7b). No difference in mononuclear cell number was observed between Control+Ethanol and DSS+Vehicle, however, DSS+Ethanol exhibited a significant increase compared to these two groups. To better characterize the cell populations present in the livers of these mice, we assessed RNA expression of markers for T-cell (*Cd3e)*, neutrophil (*Ly6g*) and macrophage (*F4/80*) lineages. The results from this analysis revealed an increase in neutrophils and macrophages after DSS administration and this was not found to be different when ethanol was added (Figure S3a). T-cells were noted to be reduced in DSS+Vehicle compared to Control+Vehicle.

Liver damage was assessed by quantifying plasma levels of alanine aminotransferase (ALT), which were found to be significantly higher in DSS+Ethanol compared to the DSS+Vehicle (Figure 7c). Liver inflammation was measured by RNA expression of various inflammatory markers. DSS+Vehicle livers had significant increases in most inflammatory markers (*Tnf-α, Cxcl1, S100a9, Tlr4, Nos2*) compared to control groups and DSS+Ethanol (Figures 7d and S3b), which was also observed at the protein level for CXCL1 (Figure S3c). The liver RNA expression of these genes in the DSS+Ethanol group were often significantly lower than the DSS+Vehicle group, while maintaining an upward trend compared to control groups that was at times significant. This trend, however, did not persist for *Lipocalin-2*, (*Lcn2*) which instead was noted to be increased to the same degree in both DSS+Vehicle and DSS+Ethanol (Figure 7d).

To define whether alterations in transcriptional activation could explain these observed changes, liver homogenates were assessed for activation of the NF-κB pathway via phosphorylation of serine 536, which increases transactivation potential and is commonly stimulated endotoxin.^44^ Control+Vehicle had no detectable phosphorylated NF-κB, while a strong increase in NF-κB phosphorylation was observed in Control+Ethanol (Figure 7e). All DSS+Vehicle livers, exhibited phosphorylated NF-κB, meanwhile livers from DSS+Ethanol displayed no phosphorylated NF-κB. Similar results were seen for STAT1 phosphorylation, wherein the DSS+Vehicle livers exhibited a strong phosphorylation of STAT1, while the phosphorylation of STAT1 was in the DSS+Ethanol livers appeared similar to controls (Figure S3d). The downregulation of inflammatory gene expression and signaling seemed to suggest the presence of immune tolerance in the DSS+Ethanol livers which has been shown to rely in part on IRAK3 expression.^45^ qPCR analysis identified an increase in *Irak3* in mice receiving DSS compared to control, but there was no difference between the DSS+Vehicle and DSS+Ethanol livers (Figure 7d). Tolerance induction by alcohol has been linked to temporal component, where shortly after an alcohol binge, tolerance occurs but waiting 24 hours after an alcohol binge, a state of increased inflammation occurs.^46^ We observed that sacrifice of DSS mice 24 hours after the last gavage of vehicle or ethanol abrogated the difference in inflammatory gene expression (Figure S3e).

## Discussion

In this study, we identified that ethanol administration post-DSS induced colitis increases intestinal *Enterobacteriaceae* burden while also reducing the *Lactobacillus:Enterobacteriaceae* ratio compared to DSS colitis alone. These bacterial changes may in part explain our previously observed finding that ethanol exacerbates colitis flare.^9^ Barrier function was similarly compromised between the mice receiving DSS+Vehicle and DSS+Ethanol, however, circulating endotoxin levels were higher in the DSS+Ethanol mice. The circulating endotoxin can initiate liver inflammation, which we observed as increased liver triglycerides, mononuclear cell infiltrate, and ALT levels in the DSS+Ethanol mice. Despite these increased markers of inflammation, examination of inflammatory gene expression and transcriptional pathways showed a decrease in their expression and activation, respectively, in the DSS+Ethanol mice, suggestive of an immune tolerant phenotype.

IBD flares and colitis models are characterized by increases in intestinal *Enterobacteriaceae*,^15–17,47^ with similar increases noted in individuals with chronic alcohol consumption.^14,18,48^ We observed that the combination of colitis and ethanol increased the *Enterobacteriaceae* greater than that seen in either experimental group separately. In fact, ethanol alone did not produce any significant changes, suggesting that the presence of preexisting intestinal inflammation may be required in order for ethanol to have an effect on the *Enterobacteriaceae*. The observed increases in the *Enterobacteriaceae* are relevant as they can penetrate the mucus layer of UC patient intestines,^15,16,21^ and studies carried out in UC disease models show that this penetration occurs before the onset of intestinal tissue damage.^22,23^ Furthermore, selectively limiting the expansion of *Enterobacteriaceae* in a mouse model of colitis has been shown to decrease colitis severity.^49^

In contrast to the *Enterobacteriaceae*, lactobacilli play a beneficial role in intestinal health and are the primary constituents of numerous probiotics.^50^ Lactobacilli have been found to be decreased in UC patients,^19^ and alcohol consumers.^18^ We observed that the DSS+Ethanol experimental group had near significant decreases of *Lactobacillus* in the cecal contents compared to Control+Vehicle and Control+Ethanol but not compared to DSS+Vehicle. Using the ratio of *Lactobacillus:Enterobacteriaceae* (where higher ratios are indicative of healthier intestines),^28,29^ we observed that both control groups exhibited a high ratio, with minimal deviation from each other, while the DSS+Vehicle group had a lower ratio, and the lowest ratio was seen in the DSS+Ethanol group. The ratio changes seen between the DSS experimental groups, highlights the importance of a prior insult in order for ethanol. A similar scenario can be seen in experiments that combine ethanol and burn injury, where minimal effects can be attributed to acute ethanol intake alone, however, in the context of a burn injury there is increased intestinal inflammation.^51^ The limited effects of ethanol alone are further underscored by the limited changes in the mucins of this study and the intestinal pathology noted in this study and our previous study.^9^

Due to the association of lactobacilli with gut health, numerous studies have turned to the reintroduction of these bacteria in the setting of colitis,^52–55^ and alcohol consumption.^13,18,56,57^ One beneficial effect of lactobacilli is their ability to limit the proliferative of *Enterobacteriaceae* and other pathogenic bacteria.^24–27^ Lactobacilli accomplishes this defense in part through the production of lactic acid that alters pH, which the lactobacilli can tolerate but other bacteria cannot.^58^ Furthermore, reduced pH enhances bacterial production of short-chain fatty acid production which promotes intestinal health and inhibits the growth of *Enterobacteriaceae* bacteria.^59–61^ Ethanol, on the other hand, has been shown to increase fecal pH which was sharply reduced by the administration of *Lactobacillus*.^13^ Therefore, in our model of DSS-colitis, the observed decrease in lactobacilli with concomitant administration of ethanol may be one explanation for the increase in Enterobacteriaceae. Additionally, overgrowth of *Enterobacteriaceae* has been shown in a chronic model of DSS colitis with ethanol administration, which could be hindered by the addition of the antimicrobial peptide, human defensin-5.^62^

As the *Enterobacteriaceae* expand, they are more likely to induce inflammatory reactions via increased IEC interaction and endotoxin translocation, leading to downstream liver inflammation,^30,31^ and even nonalcoholic fatty liver disease.^32^ In the healthy intestine, mucins and tight junction proteins serve to limit these effects.^35,63^ However, in UC, both mucins and tight junction proteins can become dysfunctional.^37,38,64^ In active UC, serum endotoxin levels rise,^65^ which may be causal in some of the observed UC liver changes, such as increased hepatic steatosis, liver enlargement,^33^ and elevated liver enzymes.^34^ Meanwhile, ethanol has a demonstrated ability to disrupt tight junction proteins, increasing endotoxin translocation,^66–69^ which can downregulate liver inflammation, an effect that is reversed by the administration of antibiotics.^46,70^ In a similar manner, mice receiving DSS exhibit increased liver mononuclear cells which are not observed in germ-free mice receiving DSS, suggesting a bacterial origin for liver inflammation during DSS-colitis.^71^ In our model, we observed that irrespective of whether mice received DSS alone or ethanol after DSS, tight junction and mucin expression were similarly decreased. Furthermore, both of these experimental groups exhibited bacterial infiltration of the intestinal mucosa, however, increased circulating levels of endotoxin were noted to only be increased in the DSS+Ethanol mice. Although circulating endotoxin did not differ significantly in the DSS treated mice compared to the control mice, the difference in circulating endotoxin levels between the DSS+Vehicle and DSS+Ethanol may be reflective of the recovery states between the two experimental groups. Withdrawal of DSS on day 5 may allow for the DSS+Vehicle mice to enter a state of recovery enhancing endotoxin clearance, while this is prevented by the ethanol administration in the DSS+Ethanol mice. Additionally, the endotoxin levels may be significantly decreased in the DSS+Vehicle mice secondary to the increased expression of liver Tlr4, which can facilitate endotoxin clearance.^72^

The changes in hepatic triglycerides and expression of lipid synthesis and uptake genes in our model appear to be multifactorial. Previous models of DSS and *Citrobacter rodentium* infection have shown that these livers decrease expression of *Fasn* and *Scd1*,^73^ which was similarly observed in our DSS experimental groups. The mechanism for this decrease is not entirely understood but does rely in part on increased levels of TNF-*α*.^74^ Meanwhile, ethanol has showed a potentiating effect on the expression of *Fasn* and *Cd36*,^75,76^ which can be appreciated in the livers of the DSS+Ethanol mice relative to DSS+Vehicle. Therefore, the increased concentration of hepatic triglycerides in the DSS+Ethanol mice may be a culmination of ethanol promoting expression of lipid synthesis and uptake genes, which is further exacerbated by the depression of *Tnf-α* expression.

The induction of tolerance from excessive endotoxin relies on a complex signaling network that reduces the activation of proinflammatory transcription factors such as NF-κB,^77^ and STAT1.^78^ The induction of tolerance relies in part on the increased expression of Irak3, which has been identified as a negative regulator of TLR signaling.^45^ We observed increases in Irak3 in both DSS treated groups but did not see any significant difference, suggesting other tolerizing effectors may be playing a role, such as Bcl-3, which can be induced by ethanol,^79^ and then inhibits the activity of NF-κB and STAT1.^80^ In contrast, the persistent expression of *Lcn2* we observed in DSS treated mice, had been described in kidney fibroblasts to occur via a biphasic mechanism wherein endotoxin activates TLR4, leading to early phase activation of c-Jun, followed by late phase and sustained activation of C/EBPδ.^81^

In summary, we observed that administration of ethanol post-DSS colitis leads to increased *Enterobacteriaceae*, decreased *Lactobacillus*, and a decreased *Lactobacillus:Enterobacteriaceae* ratio. These bacterial changes were associated with increased weight loss and shorter colons. In the DSS+Ethanol mice, the barrier dysfunction produced by ethanol, likely allows for a proportional increase in leakage of the *Enterobacteriaceae* endotoxin relative to the DSS+Vehicle mice as seen by the increased circulating levels of endotoxin. The result of this may explain the exacerbated intestinal pathology of the DSS+Ethanol mice. Meanwhile, the continued interaction of endotoxin with the liver increases triglyceride content, mononuclear cells, and liver damage, while also inducing an immune tolerant phenotype. Together these findings suggest that ethanol further exacerbates DSS colitis via an increase in the intestinal *Enterobacteriaceae* which likely contribute to the downstream effects including liver injury and immune tolerance.

## Methods

### Murine model of alcohol and colitis

Male C57BL/6 mice (8–9 weeks old; ∼23–25 g body weight) were obtained from Charles River Laboratories (Wilmington, MA). The mouse model used in this study was previously described.^9^ Briefly, mice were randomly assigned to four experimental groups: Control+Vehicle (Ctrl+V), Control+Ethanol (Ctrl+E), Dextran sodium sulfate+Vehicle (DSS+V), Dextran sodium sulfate+Ethanol (DSS+E). Mice were administered either normal drinking water or a 2% (w/v) solution of DSS (36,000–50,000 molecular weight; MP Biomedicals) ad libitum, starting on day 0 and until day 5. On day 5, DSS was stopped and mice received a gavage of either 3 g/kg ethanol or water per day until day 7. Mice were euthanized either 3 hours or 24 hours (DSS + V + 1, DSS + E + 1) after the last gavage. Mice were weighed each day to determine percent weight change relative to day 0. Following euthanasia, the large intestine was excised and its length determined. All the animal procedures were carried out in accordance with the National Institutes of Health *Guide for the Care and Use of Laboratory Animals*. These studies were approved by the Loyola University Chicago Health Sciences Division Institutional Animal Care and Use Committee.

### Bacterial DNA isolation

Fecal pellets were collected from mice on days 5 and 6. Colon contents and cecal contents were removed from mice at euthanasia on day 7. Bacterial DNA was isolated from these samples using the QIAamp PowerFecal DNA Kit (Qiagen) according to manufacturer’s guidelines. The optional 5-minute incubation at 2–8°C was not used. Isolated DNA was quantified using a NanoDrop 2000 spectrophotometer (Thermo Fisher Scientific). Due to noted PCR inhibition in the DNA from the day 5 fecal pellets of the DSS mice, all day 5 fecal pellet DNA samples were further purified using the DNeasy PowerClean Pro Cleanup Kit (Qiagen).

### Bacterial DNA qPCR

Primers for bacterial community quantification were as follows: Total bacteria- UniF340 (ACTCCTACGGGAGGCAGCAGT) and UniR514 (ATTACCGCGGCTGCTGGC), annealing temperature 63°C; *Enterobacteriaceae*- Uni515F (GTGCCAGCAGCCGCGGTAA) and Ent826R (GCCTCAAGGGCACAACCTCCAAG), annealing temperature 67°C; *Lactobacillus*- LabF362 (AGCAGTAGGGAATCTTCCA) and LabR677 (CACCGCTACACATGGAG), annealing temperature 56°C.^82^ Six µL of DNA (0.7ng/µL for fecal pellets and large intestine contents, or 7 ng/µL cecal contents) was mixed with 2 µL of each forward and reverse primer and 10 µL of iTaq Universal SYBR Green supermix (Bio-Rad) for a total reaction volume of 20 µL. Reactions were performed on a Step One Plus qPCR instrument (Applied Biosystems) and run as follows: 95°C for 3 minutes, 40 cycles of 95°C for 15 seconds, followed by data collection at the annealing temperature for 1 minute. This was followed by a melt-curve analysis. To interpret bacterial DNA relative quantity (RQ), Ct values from target bacteria (*Enterobacteriaceae* or *Lactobacillus*) were subtracted from total bacteria Ct values to obtain a ΔCt, this was used for the 2^(-ΔCt) calculation. The 2^(-ΔCt)^ value was then log_10_ transformed. For ratio assessment, the *Lactobacillus* 2^(-ΔCt)^ value was divided by the *Enterobacteriaceae* value and the result was then log_10_ transformed.

### Large IEC isolation

Isolation of large IECs was performed as described previously.^83^ The large intestine was opened longitudinally and placed in cold PBS containing a 1% penicillin/streptomycin (pen/strep) cocktail (Corning). The tissues were washed twice with PBS+pen/strep, then placed in a digestion solution (prewarmed to 37°C) containing 5% heat-inactivated fetal bovine serum (FBS), 1% HEPES, 1% pen/strep, 0.5% gentamicin, 5 mM EDTA, and 1 mM dithiothreitol in Hank’s Balanced Salt Solution. The tissues were then placed in a 37°C shaking incubator (250 rpm) for 20 minutes, then vortexed to dissociate epithelial cells and passed through a 100 μm filter set in a tube on ice. The prior step was repeated with additional digestion solution. The IECs were washed twice in PBS and then stored at −80°C until downstream processing.

### IEC RNA isolation, cDNA synthesis, and gene expression

RNA was isolated from large IECs and liver tissue using the RNeasy Mini Kit (Qiagen) according to manufacturer’s guidelines. The RNase-free-DNase Set (Qiagen) was used in conjunction with the RNA isolation kit to remove genomic DNA. RNA was quantified using a NanoDrop 2000 spectrophotometer (Thermo Fisher Scientific). cDNA was synthesized using the High Capacity cDNA Reverse Transcription Kit (Thermo Fisher Scientific) and run on a Veriti 96-well Fast Thermocycler (Life Technologies).

Expression of mucins and tight junction proteins were assessed by qPCR using TaqMan primer probes and TaqMan Fast Advanced Master Mix (Thermo Fisher Scientific). Reactions were performed on a Step One Plus qPCR instrument (Applied Biosystems). Endogenous controls for the targets are as listed on the y-axis of figures. Targets were assessed using the 2^(-ΔCt)^ method and expressed as fold change (FC) relative to Control+Vehicle or DSS + V + 1.

### Large intestine mucin staining and histopathology

A 1 cm portion of the distal large intestine was removed, fixed in Carnoy’s solution (RICCA Chemical Company), and submitted to AML labs (Jacksonville, FL). Samples were embedded in paraffin and sectioned at 5 μm onto glass slides. Slides were stained with Periodic acid-Schiff (PAS) and Alcian blue, to detect neutral and acidic mucins. Images were taken on an Olympus BX43 Microscope using an Olympus DP26 camera at total magnification of 400x. These sections were then scored based on a 0–4 point scale examining exudate, epithelial damage, polymorphonuclear leukocyte invasion, submucosal edema, and necrosis. The values from each of these categories were summed to produce the combined histopathology score.

### Endotoxin assay

Isolated plasma was tested in duplicate for endotoxin levels using the Pierce Chromogenic Endotoxin Quant Kit (Thermo Fisher Scientific), according to the manufacturer’s guidelines.

### Liver histology and oil red O staining

Livers were removed at euthanasia and divided for fixation in 10% neutral buffered formalin or embedded in optimal cutting temperature media. Formalin fixed samples were submitted to AML labs (Jacksonville, FL). Samples were embedded in paraffin and sectioned at 5 μm onto glass slides, followed by staining with hematoxylin and eosin (H&E). Images were taken on an Olympus BX43 Microscope using an Olympus DP26 camera at total magnification of 400x. For mononuclear cell quantification of H&E stained liver slides (n = 6–8 per experimental group), a single 400x area was counted by a blinded technician.

Livers embedded in optimal cutting temperature media were cut at 5 μm sections in a cryostat. Sections were stained with Oil Red O at the Loyola Histology Core. Assessment of slides was performed by a blinded pathologist. Images were taken on an Olympus BX43 Microscope using an Olympus DP26 camera at total magnification of 400x.

### Cxcl1 ELISA

Liver homogenates were assayed in duplicate using the Cxcl1 ELISA (R&D Systems) according to manufacturer’s instructions and then normalized to total protein in the liver homogenate as quantified by the DC Protein Assay (Bio-Rad).

### Fluorescent in-situ hybridization

Hybridization of distal large intestine slides with bacterial probes was completed as described previously.^84^ Briefly, unstained slides were deparaffinized in 3 washes with xylene followed by 3 washes in ethanol. Slides were dried in an incubator at 50°C for 25 minutes. Slides were incubated overnight at 50°C with 1 ng/µL of probe targeting all bacteria (EUB338: Alexa 555 5’-GCTGCCTCCCGTAGGAGT −3’) (Invitrogen) in buffer (0.9 M NaCl, 20mMTris-HCL, pH 7.5, 0.1% SDS). To wash unbound probe, the slides were incubated 15 minutes in buffer (0.9 M NaCl, 20mMTris-HCL, pH 7.5, 0.1% SDS) three times. Slides were then air-dried, mounted, and counterstained with ProLong Gold Antifade Mountant with DAPI (Thermo Fisher Scientific). Slides were imaged on a Zeiss Axiovert 200 m fluorescent microscope at total magnification of 400x and processed by the Axiovision software. In Adobe photoshop, a histogram stretch was employed for the blue and red channels to spread the image intensities across the entire intensity display range.

### Triglyceride quantification

Liver triglycerides were quantified using the Triglyceride Colorimetric Assay Kit (Cayman) according to manufacturer’s guidelines. Briefly, livers were weighed prior to homogenization in the NP40 substitute assay reagent with 1x Halt Protease and Phosphatase Inhibitor Cocktail (Thermo Fisher Scientific) and then assayed in duplicate. Data are displayed as triglyceride mg/g of liver tissue.

### Plasma ALT quantification

Plasma levels of ALT were assayed in duplicate using the Alanine Transaminase Colorimetric Activity Assay Kit (Cayman), according to manufacturer’s guidelines.

### Liver western blot

Thirty mg of liver were homogenized in 1x Cell Lysis Buffer (Cell Signaling Technologies) supplemented with 1x Halt Protease and Phosphatase Inhibitor Cocktail (Thermo Fisher Scientific). Protein was quantified using the DC Protein Assay (Bio-Rad). Protein was separated using SDS- PAGE using Bolt Bis-Tris gels (Thermo Fisher Scientific) and then transferred to a PVDF Membrane using a wet transfer method. Membrane was blocked with milk, washed with TBS, then incubated overnight with respective antibodies (pNF-κB, Cell Signaling Technologies; NF-κB Cell Signaling Technologies; pSTAT1 Abcam; STAT1 Abcam; β-actin Cell Signaling Technologies). After washing with TBST, the membrane was incubated with HRP-linked secondary antibody (Cell Signaling Technology) and developed using Western Lightning Plus-ECL, Enhanced Chemiluminescence Substrate (Perkin Elmer). Membrane was imaged on a ChemiDoc (Bio-Rad) and densitometry analysis performed using ImageLab software (Bio-Rad). Afterward the blot was stripped using Restore Plus Stripping Buffer (Thermo Fisher Scientific) and then reprobed with a different antibody.

### Statistics

Linear regression, Pearson correlation, One-way ANOVA followed by Tukey posthoc test, and student’s t-test were performed using GraphPad Prism 8 software. When values between DSS+Vehicle and DSS+Ethanol did not achieve significance, a student’s t-test was performed. In figures, the t-test appears as a dashed line. For the plasma ALT quantification, a Grubb’s test was used to identify and remove a significant outlier in the DSS+Vehicle experimental group. Statistical significance was defined as a *p* value < .05.

## Supplementary Material

Supplemental Material
