# Supplementary material for: Alcohol decreases intestinal ratio of Lactobacillus to Enterobacteriaceae and induces hepatic immune tolerance in a murine model of DSS-colitis
Source: Gut Microbes. 2020 Nov 12;12(1):1838236. doi: 10.1080/19490976.2020.1838236 (PMC7671045; doi:10.1080/19490976.2020.1838236)
Supplement: Supplemental Material [file KGMI_A_1838236_SM9358.pdf]

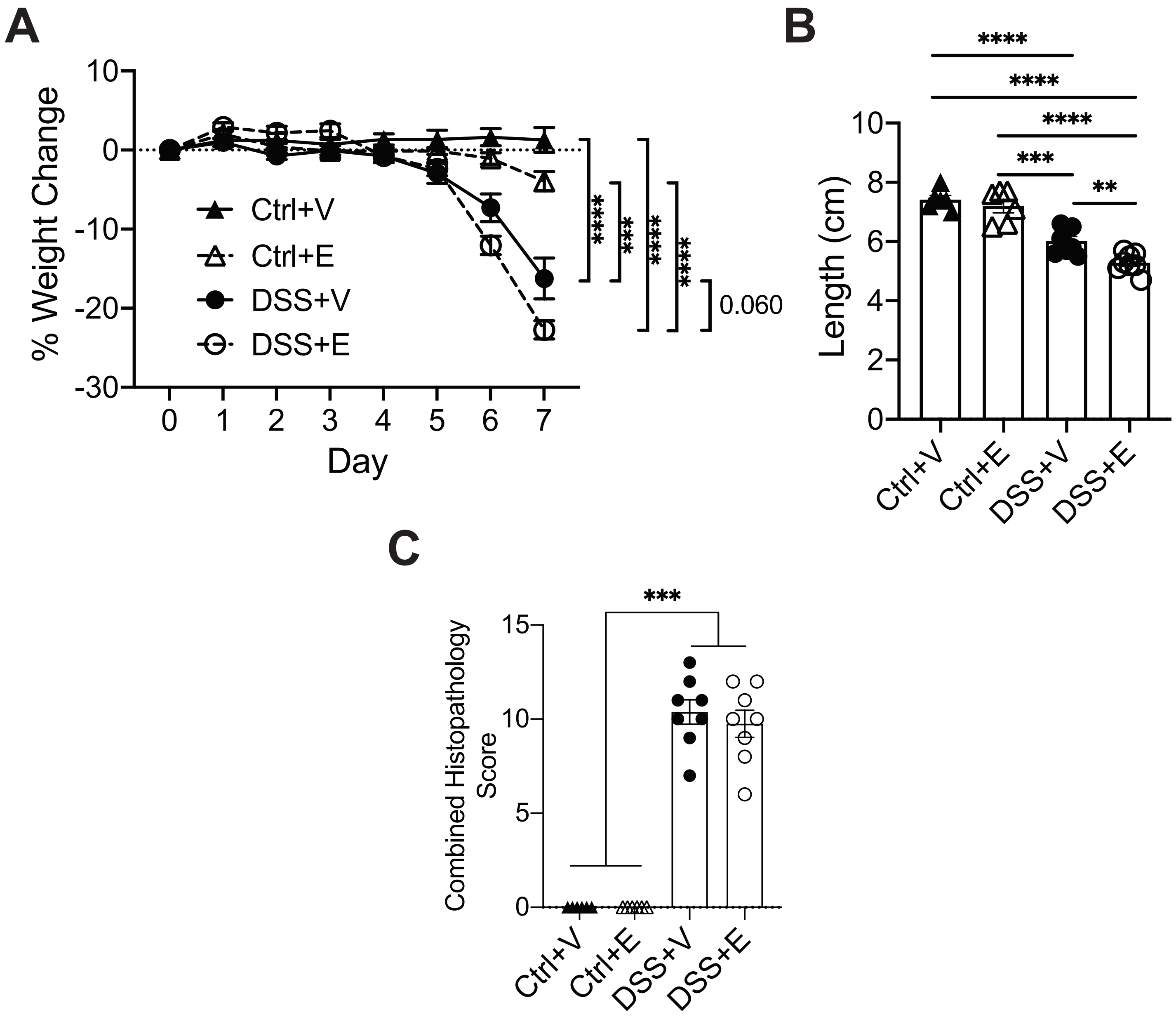

Figure S1. Increased Weight Loss and Shorter Colons in DSS+Ethanol Mice. (A) Mouse % weight change per experimental group, normalized to weight on day 0. Symbols represent the mean ± SEM for the experimental group. (B) Mouse colon length after euthanasia on Day 7, 3 hours after the last gavage. (C) Combined histopathology score for the large intestine. Bars displays mean ± SEM, with each symbol representing data from one mouse. Statistics by One-Way ANOVA with Tukey post-hoc test. n= 6-8 per group. ** $p<0.01$, *** $p<0.001$, **** $p<0.0001$.

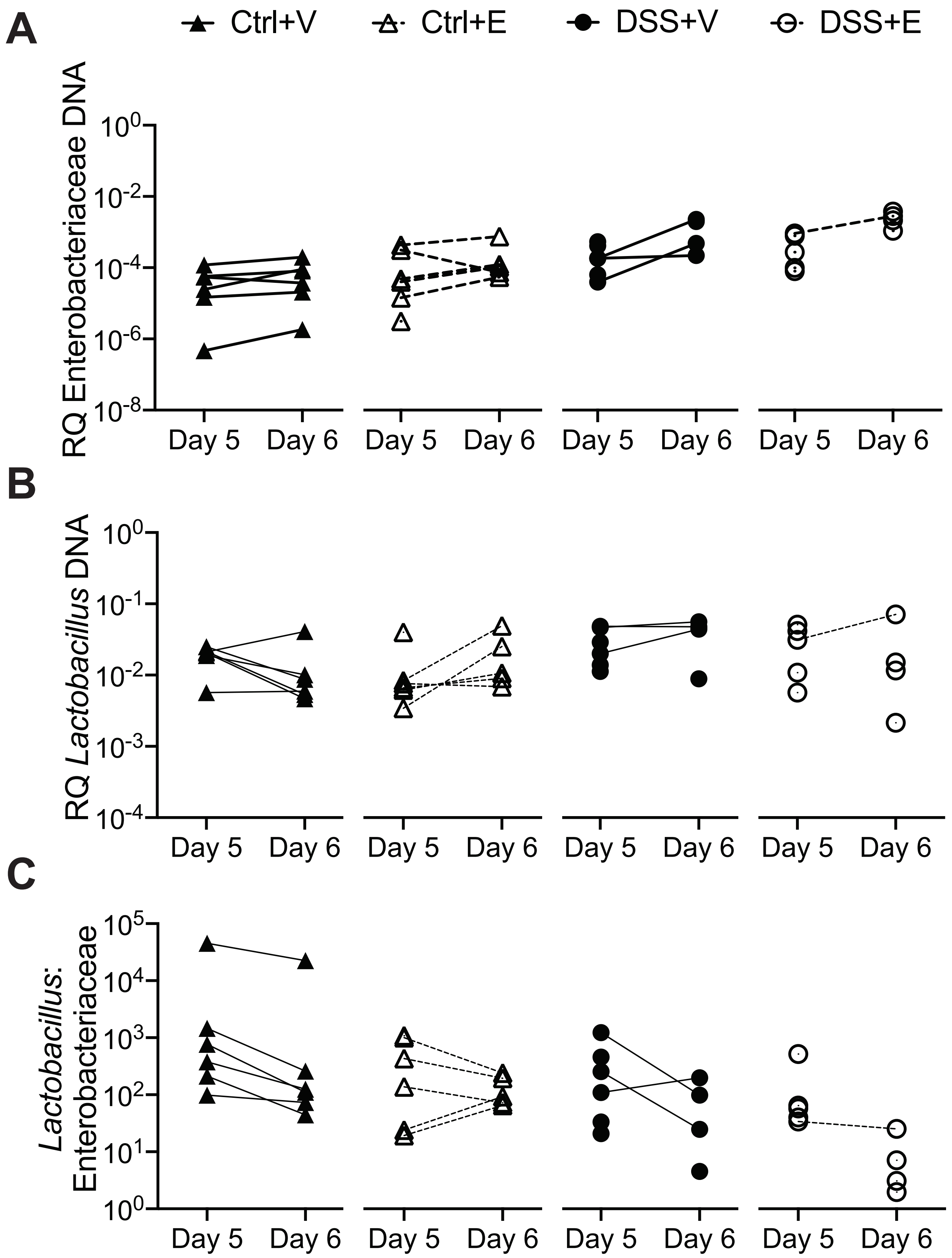

Figure S2. Enterobacteriaceae, *Lactobacillus*, and *Lactobacillus*:Enterobacteriaceae per Individual Samples in Fecal Pellets from Day 5 to Day 6. Fecal pellets were collected from mice after weighing and prior to gavage followed by DNA isolation, qPCR, and determination of bacterial RQ for (A) Enterobacteriaceae, (B) Lactobacillus, (C) Lactobacillus: Enterobacteriaceae. Each symbol represents data from one mouse with consecutive sampling from the same mouse indicated by a connecting line. RQ: Relative Quantity. n=4-6 per group.

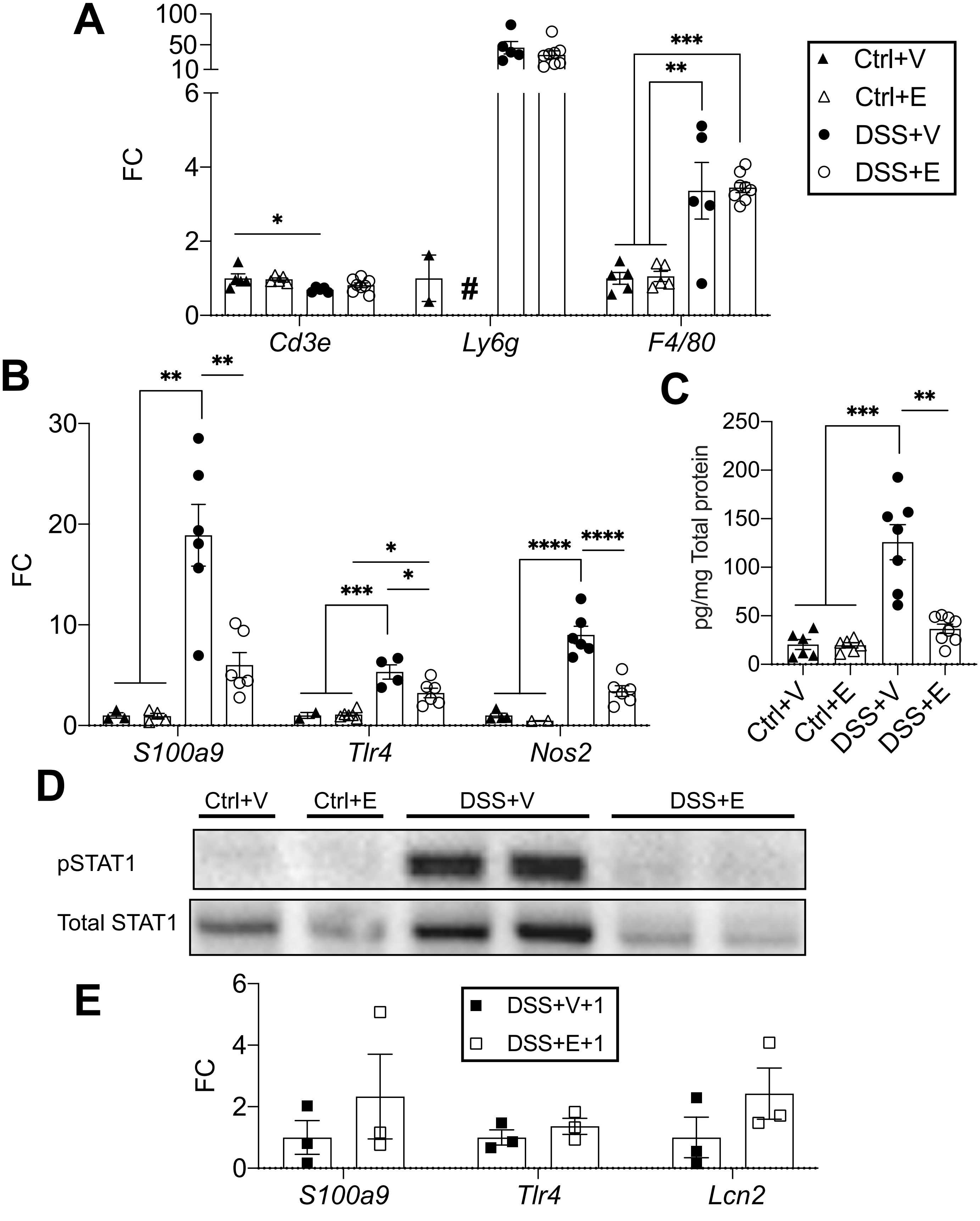

Figure S3. Hepatic Immune Cell Profiling, Additional Inflammatory Gene Expression, and STAT1 Phosphorylation. (A) Hepatic gene expression for infiltrating immune cells. # For Ly6g, all the Control+Ethanol samples were below the threshold for detection. n= 2-8 per group. (B) Hepatic inflammaotry gene expression n=2-6 per group (C) Cxcl1 protein within liver homogenates. n=6-8 per group. (D) Hepatic pStat1 and total Stat1 western blot. (E) Hepatic inflammaotry gene expression 24 hours after the last gavage of vehicle (DSS+V+1) or ethanol (DSS+E+1). n=3 per group. Bars displays mean ± SEM, with each symbol representing data from one mouse. FC: Fold Change. Statistics by One-Way ANOVA with Tukey post-hoc test. * p<0.05 ** p<0.01, *** p<0.001, **** p<0.0001.
